# Predictors of clinical response to extrafine and non-extrafine particle inhaled corticosteroids in smokers and ex-smokers with asthma

**DOI:** 10.1186/s12931-018-0961-2

**Published:** 2018-12-18

**Authors:** Fajri Gafar, Ilse M. Boudewijn, Claire A. Cox, Judith M. Vonk, Siebrig Schokker, Anne J. Lexmond, Henderik W. Frijlink, Paul Hagedoorn, Dirkje S. Postma, Maarten van den Berge

**Affiliations:** 1 Department of Pulmonary Diseases, University of Groningen, University Medical Center Groningen, PO Box 30.0001, 9700 RB Groningen, The Netherlands; 2 Groningen Research Institute for Asthma and COPD, University of Groningen, University Medical Center Groningen, PO Box 30.0001, 9700 RB Groningen, The Netherlands; 3 Department of Epidemiology, University of Groningen, University Medical Center Groningen, PO Box 30.0001, 9700 RB Groningen, The Netherlands; 4 Department of Pulmonary Diseases, Martini Hospital Groningen, PO Box 30.033, 9700 RB Groningen, The Netherlands; 5 Department of Pharmaceutical Technology and Biopharmacy, University of Groningen, Antonius Deusinglaan 1, 9713 AV Groningen, The Netherlands

**Keywords:** Asthma, Inhaled corticosteroids, Extrafine particle, Non-extrafine particle, Small airways, Smoking

## Abstract

We performed a post-hoc analysis of the OLiVIA-study investigating whether current and ex-smoking asthmatics with small airways dysfunction (SAD) show a better response in airway hyperresponsiveness (AHR) to small particle adenosine after treatment with extrafine compared to non-extrafine particle inhaled corticosteroids (ICS), and to investigate which clinical parameters predict a favorable response to both treatments. We show that smoking and ex-smoking asthmatics with and without SAD have a similar treatment response with either extrafine or non-extrafine particle ICS. We also found that lower blood neutrophils are associated with a smaller ICS-response in smokers and ex-smokers with asthma, independent from the level of blood eosinophils.

**Electronic supplementary material:**

The online version of this article (10.1186/s12931-018-0961-2) contains supplementary material, which is available to authorized users.

Asthma patients who smoke experience more severe symptoms as well as airflow limitation, and benefit less from treatment with inhaled corticosteroids (ICS) compared with non-smoking asthmatics [1]. Additionally, smoking is associated with small airways dysfunction (SAD) [2]. The small airways, defined by a diameter ≤ 2 mm contribute to the resistance in the airways of patients with obstructive airways disease [3]. This has a clinical impact since small airways can be inflamed in asthma and hence narrowed [4]. We hypothesized that smokers and ex-smokers with asthma would benefit more from extrafine than non-extrafine particle ICS. However, our OLiVIA-study showed that extrafine and non-extrafine particle ICS were equally effective in improving small airways function in current and ex-smokers with asthma [5]. This outcome might be ascribed to the fact that the presence of SAD was not an inclusion criterion in our study. Therefore, we performed a post-hoc analysis to investigate whether current and ex-smoking asthmatics with SAD show a better clinical response to extrafine compared to non-extrafine particle ICS. Next, we investigated which clinical parameters, apart from the presence of SAD, predict a favorable response to extrafine and non-extrafine particle ICS.

The OLiVIA-study was an open-label, randomized, three-way crossover, two-center study, comparing two-week treatment with extrafine hydrofluoroalkane (HFA)-beclomethasone 200 μg b.i.d. (QVAR) to non-extrafine HFA-beclomethasone 400 μg b.i.d. (Clenil) and non-extrafine HFA-fluticasone 250 μg b.i.d. (Flixotide) [5]. The primary outcome was the change in airway hyperresponsiveness (AHR) to small particle adenosine, expressed as the provocative dose of adenosine causing a 20% drop in FEV_1_ (forced expiratory volume at 1 s) from baseline to post-treatment (ΔPD_20_). Small particle adenosine is a provocative agent that acts indirectly via the release of mediators from inflammatory cells. It was chosen rather than methacholine as it is a more sensitive measurement to detect improvement in AHR after treatment with ICS in patients with asthma [6].

Since there is no clearly defined golden standard for SAD, we applied various parameters next to the PD_20_ adenosine, i.e. parameters of spirometry, body plethysmography, impulse oscillometry (IOS) and multiple breath nitrogen washout (MBNW). We used cut-off values for SAD parameters as follows: forced expiratory flow between 25 and 75% of forced vital capacity [FEF_25–75_] < lower limit of normal (LLN) from spirometry; ratio of residual volume to total lung capacity [RV/TLC] > upper limit of normal (ULN) from body plethysmography; difference between resistance at 5 Hz and 20 Hz [R_5_-R_20_] > 0.1 kPa sL^− 1^ from IOS [7]; and ventilation heterogeneity of the acinar structures [S_acin_] and conductive airways [S_cond_] > ULN from MBNW [8]. These measurements were taken at the baseline visit after an ICS washout period of four to six weeks. The presence of SAD was defined as: at least 3 out of 5 criteria fulfilled when all lung function measurements were performed, or with 2 out of 3 criteria if MBNW was not carried out, which was the case in 8 patients.

We performed a two-sided paired t-test or Wilcoxon test to assess the difference in ΔPD_20_ adenosine after treatment with extrafine compared to non-extrafine particle ICS in patients with SAD and patients without SAD. PD_20_ values were log_2_-transformed prior to analyses. To investigate the difference in ΔPD_20_ between patients with SAD and without SAD within one treatment group, we performed a student t-test or Mann-Whitney test. Next, we explored which baseline variables (i.e. demographics, small and large function measures, presence of atopy and blood leukocyte counts) were associated with ΔPD_20_ adenosine after extrafine and non-extrafine particle ICS treatment using univariate linear regression analysis. We finally performed multivariate linear regression analyses for each treatment type to identify independent clinical predictors for ΔPD_20_ including age, sex, smoking status, numbers of neutrophils and eosinophils, and completed with those variables showing a trend towards association with ΔPD_20_ (*p* < 0.1) in the univariate analysis. We allowed a maximum of 7 variables to be included in the multivariate model. The variables in this final model were selected based on the following criteria: (1) the variable changed regression coefficients (B) > 10%, (2) the model had highest total explained variance (R^2^), and (3) the model preferably included the highest number of subjects.

We analyzed 43 asthma patients (22 smokers and 21 ex-smokers), 42% being male, with mean (standard deviation (SD)) age 45 (12.6) years, FEV_1_ 83 (14.5) % predicted, and PD_20_ 2.69 (3.41) mg. Baseline characteristics of the study population are presented in Additional file 1: Table S1. At baseline, we found that SAD was present in 18 (42%) patients (see Additional file 1: Table S2). Asthma patients with and without SAD had similar response in PD_20_ adenosine to extrafine (QVAR) and non-extrafine particle ICS treatments (Clenil and Flixotide) (Fig. 1; and Additional file 1: Table S3).

**Fig. 1:**
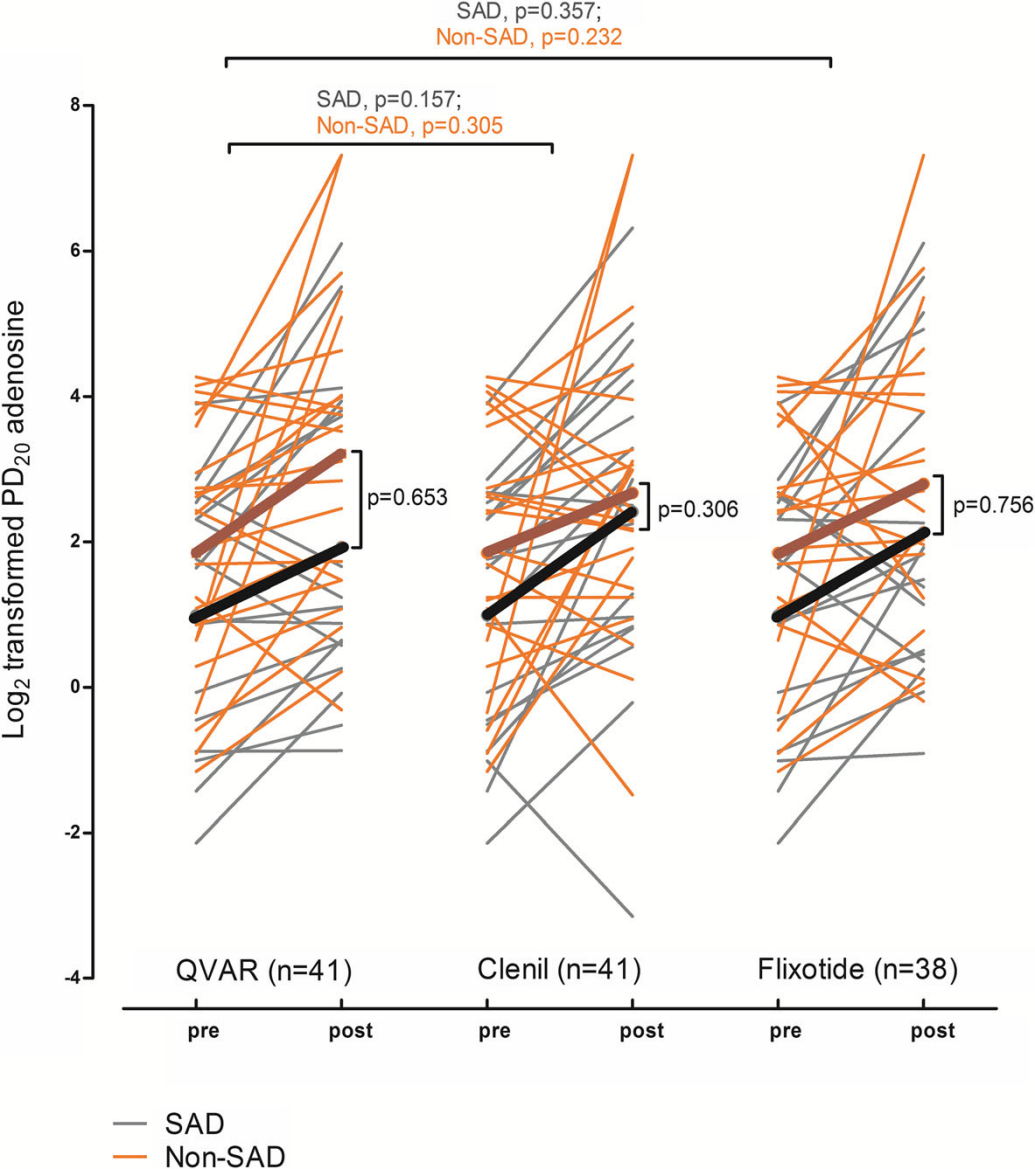
Change in PD_20_ adenosine from baseline to post-treatment with QVAR, Clenil and Flixotide in patients with and without small airways dysfunction (SAD). Each line depicts a subject while the bold line depicts the mean change in PD_20_ adenosine in response to the treatment. PD_20_: provocative dose of small particle adenosine causing a 20% drop in forced expiratory volume at 1 s (FEV_1_)

Results of the univariate linear regression analyses are presented in Additional file 1: Table S4. Multivariate linear regression analyses (Table 1) showed that no parameters of SAD were associated with treatment response to either small or large particle ICS. Lower numbers of blood neutrophils were associated with a larger increase in PD_20_ adenosine, independently from the level of blood eosinophils, in patients treated with QVAR, Clenil and Flixotide (all *p* < 0.05). Higher blood eosinophils were also associated with a larger increase in PD_20_ adenosine in patients treated with Flixotide (*p* < 0.05). Finally, younger age tended to be associated with a larger increase in PD_20_ adenosine after using Clenil (*p* = 0.05).

**Table 1:** Multivariate linear regression analysis of baseline variables with change in airway hyperresponsiveness to small particle adenosine (ΔPD_20_) as dependent variable in patients treated with QVAR, Clenil and Flixotide

	Baseline predictors	B	95% CI	*p*-value	R^2^
ΔPD_20_ QVAR	Age, years	−0.10	− 0.06; 0.04	0.703	0.64
	Sex, male/ female	0.41	−0.78; 1.60	0.485	
	Current smoking, yes/no	−0.47	−1.87; 0.93	0.493	
	Blood neutrophils, 10^9^/L	−0.55	−1.00; − 0.09	0.020	
	Blood eosinophils, 10^9^/L	2.81	−0.48; 6.09	0.090	
	FEV_1_, % predicted	0.04	−0.01; 0.09	0.090	
	LCI at 2.5%	−0.30	− 0.69; 0.08	0.117	
ΔPD_20_ Clenil	Age, years	−0.06	− 0.12; 0.00	0.050	0.48
	Sex, male/ female	0.80	−0.42; 2.03	0.192	
	Current smoking, yes/no	−0.95	−2.33; 0.43	0.170	
	Blood neutrophils, 10^9^/L	−0.61	−1.04; − 0.18	0.006	
	Blood eosinophils, 10^9^/L	3.04	−0.06; 6.15	0.054	
	FEV_1_, % predicted	0.03	−0.01; 0.07	0.180	
ΔPD_20_ Flixotide	Age, years	−0.03	−0.08; 0.02	0.181	0.60
	Sex, male/ female	0.85	−0.25; 1.96	0.125	
	Current smoking, yes/no	−0.33	−1.51; 0.85	0.573	
	Blood neutrophils, 10^9^/L	−0.78	−1.13; −0.43	0.000	
	Blood eosinophils, 10^9^/L	3.53	0.88; 6.17	0.011	
	FEV_1_, % predicted	0.002	−0.04; 0.04	0.937	
	RV/TLC, % predicted	−0.03	−0.06; 0.004	0.089	

B: regression coefficients; CI: Confidence Intervals; R^2^: total explained variance; ΔPD_20_: the change from baseline to post-treatment in the provocative dose of small particle adenosine causing a 20% drop in FEV_1_; FEV_1_: forced expiratory volume in 1 s; LCI: lung clearance index; and RV/TLC: the ratio of residual volume to total lung capacity

We did not confirm our hypothesis that current and ex-smoking asthmatics with SAD had a better treatment response to QVAR compared to Clenil and Flixotide. In line with previous reports on better clinical effects of ICS in asthmatics with eosinophilia [9–11], we show that higher blood eosinophils are associated with a less severe AHR after treatment with non-extrafine particle ICS (Flixotide). Of interest, our study shows that higher blood neutrophils are associated with more severe AHR after treatment with both extrafine and non-extrafine particle ICS in smokers and ex-smokers with asthma, independently from the level of blood eosinophils. Findings by Telenga et al. support that lower blood neutrophils are associated with an increase in FEV_1_ after 2-week ICS-therapy [11]. Taken together, we find that higher blood neutrophils are associated with less clinical ICS-response in smokers and ex-smokers with asthma. However, our data (Additional file 1: Figure S1) do not clearly show a cut-off value for eosinophilia or neutrophilia that can be used in clinical practice to define a positive response. Future studies have to assess whether this change in inflammation in peripheral blood is reflected by changes in the small and larger airways.

A strength of the study is that we defined SAD by cut-off levels applying multiple measurement techniques including spirometry, body plethysmography, impulse oscillometry and multiple breath nitrogen washout. A limitation is the relatively small sample size of patients with SAD and non-SAD which limits the power of the study and raises the risk of type I error. However, a post-hoc power analysis indicated that we had a sufficient number of patients to detect a difference of one doubling dose increase in PD_20_ adenosine between patients with and without SAD. With a standard deviation of one doubling dose, β = 0.8 and α = 0.05, we would need 16 patients per group while in our study, 18 patients with and 23 patients without SAD were investigated. Further studies with larger sample sizes would provide a clearer picture of SAD in smokers and ex-smokers with asthma. Another possible limitation is that we treated for two weeks which may have been too short to improve SAD.

In conclusion, we show that smoking and ex-smoking asthmatics with and without SAD have a similar response to small particle adenosine after treatment with either extrafine or non-extrafine particle ICS. These findings suggest that clinicians may not need to consider SAD in order to decide whether current- and ex-smoking asthmatics would benefit preferentially from treatment with extrafine rather than non-extrafine particle ICS. Of importance, we find that lower blood neutrophils is a favorable predictor of ICS response, independent from the level of blood eosinophils.

## Additional file

Additional file 1: **Table S1.** Baseline characteristics. **Table S2.** Baseline values of measurements of small airways dysfunction. **Table S3.** Difference in ΔPD20 adenosine in patients with and without small airways dysfunction after treatment with QVAR, Clenil and Flixotide. **Table S4.** Univariate linear regression analysis of baseline variables and change in airway hyperresponsiveness (ΔPD20) in patients treated with QVAR, Clenil and Flixotide. **Figure S1.** Scatterplots of significant correlations between baseline variables and ΔPD20 adenosine. (PDF 731 kb)

## Abbreviations

ΔPD_20_: The change from baseline to post-treatment in the provocative dose of small particle adenosine causing a 20% drop in forced expiratory volume in 1 s (FEV_1_)
AHR: Airway hyperresponsiveness
AX: The area under curve of reactance
BMI: Body mass index
CFC: Chlorofluorocarbon
FEF_25–75_: Forced expiratory flow between 25 and 75% of FVC
FVC: Forced vital capacity
F_res_: Resonance frequency
HFA: Hydrofluoroalkane
ICS: Inhaled corticosteroids
LCI: Lung clearance index
LLN: Lower limit of normal
R_5_-R_20_: The difference between resistance at 5 Hz and 20 Hz
RV: Residual volume
RV/TLC: The ratio of residual volume to total lung capacity
SAD: Small airways dysfunction
S_acin_: Ventilation heterogeneity of the acinar structures
S_cond_: Ventilation heterogeneity of the conductive airways
TLC: Total lung capacity
ULN: Upper limit of normal
X_5_: The reactance at 5 Hz

## References

[1] Thomson NC, Spears M (2005). The influence of smoking on the treatment response in patients with asthma. Curr Opin Allergy Clin Immunol..

[2] Verbanck S, Schuermans D, Meysman M, Paiva M, Vincken W (2004). Noninvasive assessment of airway alterations in smokers: the small airways revisited. Am J Respir Crit Care Med..

[3] van der Wiel E, ten Hacken NH, Postma DS, van den Berge M (2013). Small-airways dysfunction associates with respiratory symptoms and clinical features of asthma: a systematic review. J Allergy Clin Immunol..

[4] Kraft M, Pak J, Martin RJ, Kaminsky D, Irvin CG (2001). Distal lung dysfunction at night in nocturnal asthma. Am J Respir Crit Care Med..

[5] Cox CA, Boudewijn IM, Vroegop SJ, Schokker S, Lexmond AJ, Frijlink HW (2017). Extrafine compared to non-extrafine particle inhaled corticosteroids in smokers and ex-smokers with asthma. Respir Med..

[6] van den Berge M, Meijer RJ, Kerstjens HA, de Reus DM, Koeter GH, Kauffman HF (2001). PC(20) adenosine 5′-monophosphate is more closely associated with airway inflammation in asthma than PC(20) methacholine. Am J Respir Crit Care Med.

[7] Manoharan A, Anderson WJ, Lipworth J, Lipworth BJ (2015). Assessment of spirometry and impulse oscillometry in relation to asthma control. Lung..

[8] Verbanck S, Thompson BR, Schuermans D, Kalsi H, Biddiscombe M, Stuart-Andrews C (2012). Ventilation heterogeneity in the acinar and conductive zones of the normal ageing lung. Thorax..

[9] Szefler SJ, Martin RJ, King TS, Boushey HA, Cherniack RM, Chinchilli VM (2002). Significant variability in response to inhaled corticosteroids for persistent asthma. J Allergy Clin Immunol..

[10] Meijer RJ, Postma DS, Kauffman HF, Arends LR, Koeter GH, Kerstjens HA (2002). Accuracy of eosinophils and eosinophil cationic protein to predict steroid improvement in asthma. Clin Exp Allergy..

[11] Telenga ED, Kerstjens HA, Ten Hacken NH, Postma DS, van den Berge M (2013). Inflammation and corticosteroid responsiveness in ex-, current- and never-smoking asthmatics. BMC Pulm Med..

