# Supplementary material for: Predictors of clinical response to extrafine and non-extrafine particle inhaled corticosteroids in smokers and ex-smokers with asthma
Source: Respir Res. 2018 Dec 18;19:256. doi: 10.1186/s12931-018-0961-2 (PMC6299530; doi:10.1186/s12931-018-0961-2)
Supplement: Supplementary file 1 — Table S1. Baseline characteristics. Table S2. Baseline values of measurements of small airways dysfunction. Table S3. Difference in ΔPD20 adenosine in patients with and without small airways dysfunction after treatment with QVAR, Clenil and Flixotide. Table S4. Univariate linear regression analysis of baseline variables and change in airway hyperresponsiveness (ΔPD20) in patients treated with QVAR, Clenil and Flixotide. Figure S1. Scatterplots of significant correlations between baseline variables and ΔPD20 adenosine. (PDF 731 kb) [file 12931_2018_961_MOESM1_ESM.pdf]

Additional file 1:

Table S1. Baseline characteristics

	Total	Smokers	Ex-smokers
	n=43	n=22	n=21
Male, n (%)	18 (42)	9 (41)	9 (43)
Age, years	44.98 (12.64)	39.55 (12.01)	50.67 (10.84)
Presence of atopy, n (%)	27 (64)	14 (67)	13 (62)
Number of packyears*	16.80 (11.00; 24.00)	20.00 (11.75; 29.44)	14.00 (10.00; 23.00)
BMI, kg m^{-2}	26.54 (5.62)	25.83 (6.14)	27.27 (5.08)
Waist/hip ratio	0.91 (0.10)	0.88 (0.08)	0.93 (0.12)
Blood neutrophils (10^9/L)	4.50 (1.73)	5.25 (1.71)	3.62 (1.32)
Blood eosinophils (10^9/L)	0.29 (0.20)	0.35 (0.22)	0.24 (0.18)
Total IgE, kIU/L*	107 (23.97; 483.75)	138 (20.60; 548.50)	89 (22.95; 321.0)
PD_{20} adenosine, mg	2.69 (3.41)	2.38 (3.25)	3.07 (3.66)
FEV_1, % predicted	83 (14.47)	83 (11.98)	84 (16.99)
FEV_1/FVC, %	67 (10.24)	70 (10.84)	65 (9.31)
$FEF_{25-75,}$ % predicted	49 (22.56)	52 (22.22)	45 (22.87)
TLC, % predicted	105 (12.22)	103 (13.58)	107 (10.53)
RV, % predicted	115 (27.57)	113 (28.81)	117 (26.81)
RV/TLC, % predicted	102 (18.96)	103 (17.79)	102 (20.55)
R_5-R_{20}, kPa sL^{-1}	0.13 (0.13)	0.11 (0.13)	0.15 (0.13)
X_5, kPa sL^{-1}*	-0.14 (-0.21; -0.09)	-0.14 (-0.21; -0.08)	-0.13 (-0.22; -0.10)
F_{res}, Hz	17.19 (6.35)	16.49 (6.68)	17.91 (6.05)
AX, Hz kPa sL^{-1}*	0.75 (0.24; 1.82)	0.59 (0.19; 1.51)	0.94(0.29; 2.07)
LCI at 2.5%	10.21 (2.06)	9.74 (1.72)	10.63 (2.28)
S_{cond} , L^{-1}	0.04 (0.03)	0.04 (0.02)	0.04 (0.03)
$S_{acin,}$ L^{-1}	0.17 (0.09)	0.14 (0.06)	0.20 (0.11)

Data are presented as mean with standard deviation (SD), unless stated otherwise: *median with interquartile ranges (IQR); BMI: body mass index; PD_{20} adenosine: provocative dose of small particle adenosine causing a 20% drop in FEV_1; FEV_1: forced expiratory volume in 1 second; FVC: forced vital capacity; FEF_{25-75}: forced expiratory flow between 25% and 75% of FVC; TLC: total lung capacity; RV: residual volume; RV/TLC: the ratio of residual volume to total lung capacity; R_5-R_{20}: the difference between resistance at 5 Hz and 20 Hz; X_5: the reactance at 5 Hz; F_{res}: resonance frequency; AX: the area under curve of reactance; LCI: lung clearance index; S_{cond}: ventilation heterogeneity of the conductive airways; and S_{acin}: ventilation heterogeneity of the acinar structures.

Table S2. Baseline values of measurements of small airways dysfunction

	Patients with SAD	Patients without SAD
	n=18	n=25
$FEF_{25\text{-}75}$, % predicted	37 (13.20)	58 (23.95)
RV/TLC, % predicted	113 (20.89)	95 (13.71)
R_5-R_{20}, kPa sL^{-1}	0.22 (0.14)	0.06 (0.07)
S_{acin}, L^{-1*}	0.20 (0.07)	0.14 (0.09)
S_{cond}, L^{-1*}	0.06 (0.03)	0.03 (0.01)

Data are presented as mean with standard deviation (SD); SAD: small airways dysfunction; $FEF_{25\text{-}75}$: forced expiratory flow between 25% and 75% of forced vital capacity (FVC); RV/TLC: the ratio of residual volume to total lung capacity; R_5-R_{20}: the difference between resistance at 5 Hz and 20 Hz; S_{acin}: ventilation heterogeneity of the acinar structures; and S_{cond}: ventilation heterogeneity of the conductive airways.

Table S3. Difference in ΔPD_{20} adenosine in patients with and without small airways dysfunction after treatment with QVAR, Clenil and Flixotide

ΔPD_{20} adenosine	Patients with SAD		Patients without SAD		*p*-value
	n (%)	Median (IQR)	n (%)	Median (IQR)	
QVAR (n=41)	17 (41)	0.69 (-0.02; 2.07)	24 (59)	0.82 (0.06; 1.86)	0.653¥
Clenil (n=41)	18 (44)	1.73 (0.80; 2.14)	23 (56)	-0.00 (-0.75; 1.47)	0.306¥
Flixotide (n=38)	17 (45)	0.96 (0.03; 2.58)	21 (55)	0.27 (-0.59; 1.86)	0.756¥
QVAR vs Clenil	*p*=0.157£		*p*=0.305£		
QVAR vs Flixotide	*p*=0.357£		*p*=0.232£		

Data are presented as number with percentages (%) and median with interquartile ranges (IQR); *p*-values are obtained from ¥student t-test/ Mann-Whitney test and £paired t-test/ Wilcoxon test; ΔPD_{20}: the change from baseline to post-treatment in the provocative dose of small particle adenosine causing a 20% drop in forced expiratory volume in 1 second; SAD: small airways dysfunction.

Table S4: Univariate linear regression analysis of baseline variables and change in airway hyperresponsiveness (ΔPD_{20}) in patients treated with QVAR, Clenil and Flixotide.

	ΔPD_{20} QVAR (n=41)		ΔPD_{20} Clenil (n=41)		ΔPD_{20} Flixotide (n=38)	
	B	95% CI	B	95% CI	B	95% CI
Age, years	0.01	-0.04; 0.05	-0.03	-0.08; 0.02	-0.02	-0.07; 0.03
Sex, male/ female	0.45	-0.69; 1.59	1.17#	-0.14; 2.49	0.58	-0.70; 1.87
Current smoking, yes/no	-0.88	-1.99; 0.22	-1.10#	-2.41; 0.21	-0.79	-2.06; 0.48
Presence of atopy, yes/no	-0.19	-1.38; 0.99	0.17	-1.28; 1.63	-0.05	-1.43; 1.32
Number of packyears	-0.03	-0.07; 0.02	-0.06*	-0.11; -0.01	-0.03	-0.08; 0.01
BMI, kg/m^2	-0.08	-0.18; 0.02	-0.04	-0.16; 0.08	-0.12*	-0.23; -0.01
Waist/hip ratio	-1.67	-7.02; 3.68	-4.93	-11.14; 1.28	-4.07	-9.80; 1.66
Blood neutrophils, $10^9/L$	-0.48**	-0.78; -0.17	-0.37#	-0.77; 0.03	-0.55**	-0.89; -0.22
Blood eosinophils, $10^9/L$	1.00	-1.77; 3.77	2.73	-0.68; 6.13	2.32	-0.89; 5.52
Total IgE, kIU/L	0.00	-0.002; 0.001	0.001	-0.001; 0.002	0.00	-0.002; 0.001
FEV_1, % predicted	0.06**	0.02; 0.09	0.06*	0.01; 0.10	0.05**	0.01; 0.10
FEV_1/FVC, %	0.03	-0.02; 0.08	0.04	-0.02; 0.11	0.03	-0.03; 0.09
$FEF_{25-75,}$ % predicted	0.03**	0.01; 0.06	0.04*	0.01; 0.06	0.03*	0.01; 0.06
TLC, % predicted	0.03	-0.01; 0.08	0.04	-0.02; 0.09	0.01	-0.04; 0.07
RV, % predicted	-0.01	-0.03; 0.01	-0.002	-0.03; 0.02	-0.02	-0.04; 0.01
RV/TLC, % predicted	-0.03*	-0.06; -0.001	-0.01	-0.05; 0.02	-0.04*	-0.07; -0.01
R_5-R_{20}, kPa sL^{-1}	-2.67	-7.00; 1.66	-1.86	-6.98; 3.27	-3.50	-8.27; 1.28
X_5, kPa sL^{-1}	1.78	-1.73; 5.29	0.78	-3.43; 5.00	2.43	-1.43; 6.29
F_{res}, Hz	-0.06	-0.15; 0.03	-0.04	-0.14; 0.07	-0.05	-0.16; 0.04
AX, Hz kPa sL^{-1}	-0.15	-0.48; 0.18	-0.11	-0.50; 0.28	-0.20	-0.56; 0.16
LCI at 2.5%	-0.43*	-0.75; -0.11	-0.19	-0.59; 0.21	-0.46*	-0.81; -0.10
$S_{cond,}$ L^{-1}	-17.75	-44.25; 8.74	15.28	-16.77; 47.34	-7.08	-37.71; 23.54
$S_{acin,}$ L^{-1}	-5.36	-12.82; 2.10	-5.67	-14.44; 3.10	-6.13	-14.20; 1.94

Data are presented as regression coefficients (B) and 95% confidence intervals (CI); ***p*<0.01; **p*<0.05; #*p*<0.1; ΔPD_{20}: the change from baseline to post-treatment in the provocative dose of small particle adenosine causing a 20% drop in FEV1; BMI: body mass index; FEV_1: forced expiratory volume in 1 second; FVC: forced vital capacity; FEF_{25-75}: forced expiratory flow between 25% and 75% of FVC; TLC: total lung capacity; RV: residual volume; RV/TLC: the ratio of residual volume to total lung capacity; R_5-R_{20}: the difference between resistance at 5 Hz and 20 Hz; X_5: the reactance at 5 Hz; F_{res}: resonance frequency; AX: the area under curve of reactance; LCI: lung clearance index; S_{cond}: ventilation heterogeneity of the conductive airways; and S_{acin}: ventilation heterogeneity of the acinar structures.

Figure legend

Figure S1. Scatterplots of significant correlations between baseline variables and ΔPD_{20} adenosine. ΔPD_{20}: the change from baseline to post-treatment in the provocative dose of small particle adenosine causing a 20% drop in forced expiratory volume in 1 second (FEV1). Figure S1:A shows the correlation between age and ΔPD_{20} after treatment with Clenil; Figures S1:B-D show the correlation between blood neutrophils and ΔPD_{20} after treatment with QVAR, Clenil and Flixotide, respectively; Figures S1:E show the correlation of blood eosinophils and ΔPD_{20} after treatment with Flixotide

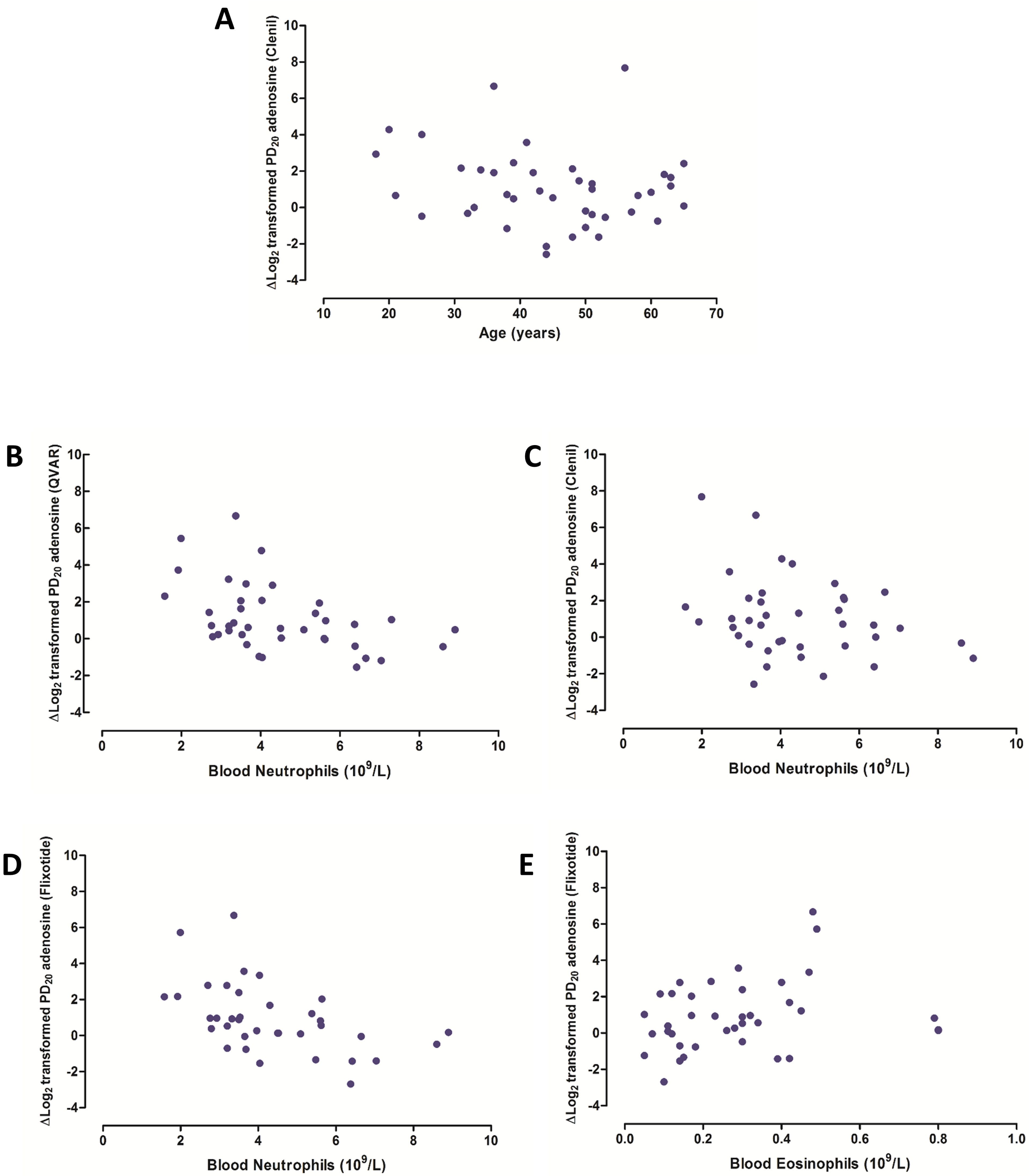
A
ΔLog2 transformed PD20 adenosine (Clenil)
Age (years)
B
ΔLog2 transformed PD20 adenosine (QVAR)
Blood Neutrophils (10^9/L)
C
ΔLog2 transformed PD20 adenosine (Clenil)
Blood Neutrophils (10^9/L)
D
ΔLog2 transformed PD20 adenosine (Flixotide)
Blood Neutrophils (10^9/L)
E
ΔLog2 transformed PD20 adenosine (Flixotide)
Blood Eosinophils (10^9/L)
